# Spondylometaphyseal dysplasia: an uncommon disease

**DOI:** 10.1590/0100-3984.2015.0159

**Published:** 2017

**Authors:** Márcio Luís Duarte, Élcio Roberto Duarte, Daniela Brasil Solorzano, Edgar Brasil Solorzano, Jael Brasil de Alcântara Ferreira

**Affiliations:** 1 WebImagem, São Paulo, SP, Brazil.; 2 Brasil Imagem Medicina Diagnóstica, Santos, SP, Brazil.

Dear Editor,

A 2-year-old female patient, born by normal delivery, without complications, at 39 weeks of gestation, all prenatal test results having been normal, was referred to the department of orthopedics and traumatology for investigation of deformities of the thorax and ankle, as well as dwarfism. She showed no psychomotor alterations. The parents of the child were healthy, with no history of malformations, and the patient was their only child. They reported that the child had been born with dental precocity, with 9 teeth at birth, and began to present changes in the thorax and ankles at 4 months of age, those changes progressing thereafter. They also reported that the child had not grown, having been 75 cm tall since the age of 1 year and the same weight, approximately 9 kg, since the age 9 months, both of those measurements, according to the US CDC, being below the 5th percentile.

**Figure 1:**
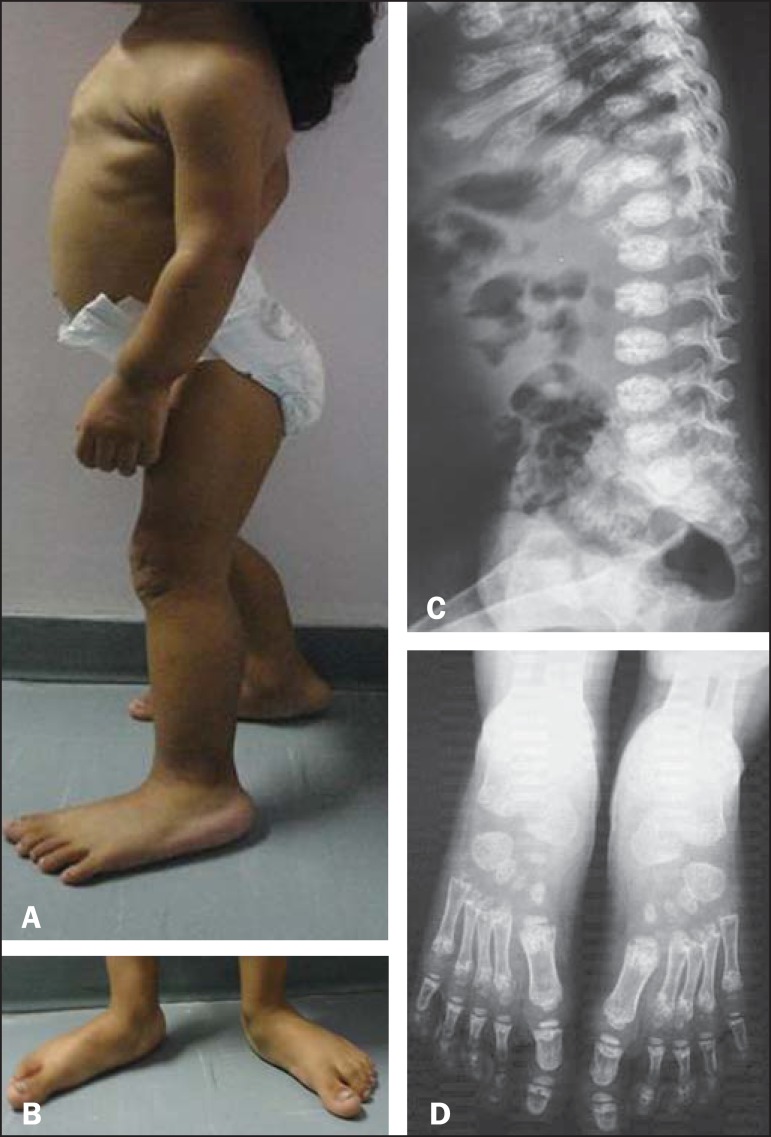
**A,B:** Physical examination showing a prominent sternum (**A**) and flat feet (**B**). **C,D:** X-rays showing platyspondyly and deformities of the ribs (**C**), as well as metaphyseal deformations such as bone rarefaction, aerated bone containing trabeculae, and cortical irregularity (**D**).

Physical examination of the patient showed a prominent sternum and shortening of the trunk, as well as discrete coxa vara with rotation to the right, flat feet, and deformity of the wrists (Figures 1A and 1B). On X-rays, we observed metaphyseal deformities such as bone rarefaction, aerated bone containing trabeculae, and cortical irregularity, as well as right-sided scoliosis and deformities of the ribs (Figures 1C and 1D). Using Todd's Atlas of Skeletal Maturation as a reference, we determined the bone age to be 21 months. Computed tomography scans (not shown) of the cervical spine and of the head, respectively, showed discrete hypoplasia of the odontoid process and a reduction in the amount white matter around the posterior horn of the lateral ventricles, neither of which have been reported in the literature.

Spondylometaphyseal dysplasia (SMD) was first described in 1967 by Kozlowski et al.^(1)^, who defined it as a rare new form of bone dysplasia comprising various types of chondrodystrophy characterized by metaphyseal irregularities of the long bones, together with generalized platyspondyly of varying severity in the spine^(1,2)^. It produces a phenotypic spectrum of disorders, genotypically being autosomal dominant^(3)^. Kozlowski-type SMD, also known as type 1 SMD, is the most common form of the disease^(1)^.

The symptoms of SMD vary depending on the age of the patients^(1)^, the principal symptoms being as follows: limited postnatal growth; rhizomelic shortening of the limbs in early childhood evolving to shortening of the trunk by the age of 10 years; thoracic hypoplasia, which causes respiratory problems in the neonatal period and increases susceptibility to respiratory tract infection^(4)^; scoliosis with dorsal kyphosis; abnormalities of the metaphyses and pelvis^(5)^; odontoid hypoplasia; and valgus of the knees and claudication^(6)^, the latter typically being the first sign of the disease^(2)^. There might be little or no ossification of the cervical vertebrae, leading to cervical instability and swan neck deformity^(7)^.

A review of the literature revealed that there are currently 10 recognized subtypes of SMD. However, there in no consensus in the medical literature regarding those subtypes, because they are based characteristics that are minimally different. Some subtypes are based on reports of only one case, and others can be diagnosed only after years of follow-up, which is difficult. For example, the longest follow-up period in a report of Sedaghatian-type SMD was 161 days. Therefore, there is no acceptable standard for subclassifying the disease.
